# Nonlinear landscape and cultural response to sea-level rise

**DOI:** 10.1126/sciadv.abb6376

**Published:** 2020-11-04

**Authors:** Robert L. Barnett, Dan J. Charman, Charles Johns, Sophie L. Ward, Andrew Bevan, Sarah L. Bradley, Kevin Camidge, Ralph M. Fyfe, W. Roland Gehrels, Maria J. Gehrels, Jackie Hatton, Nicole S. Khan, Peter Marshall, S. Yoshi Maezumi, Steve Mills, Jacqui Mulville, Marta Perez, Helen M. Roberts, James D. Scourse, Francis Shepherd, Todd Stevens

**Affiliations:** 1 Geography, University of Exeter, Amory Building, Rennes Drive, Exeter EX4 4RJ, UK.; 2 Département de biologie, chimie et géographie & Centre for Northern Studies (CEN), Université du Québec à Rimouski, Rimouski, Québec, Canada.; 3 Heritage Consultant, Sunset, Trewennack, Helston, Cornwall TR13 0PL, UK.; 4 Centre for Applied Marine Sciences, School of Ocean Sciences, Bangor University, Menai Bridge, Isle of Anglesey LL59 5AB, UK.; 5 UCL Institute of Archaeology, University College London, 31-34 Gordon Square, London WC1H 0PY, UK.; 6 Department of Geography, University of Sheffield, Winter Street, Sheffield S3 7ND, UK.; 7 Cornwall and Isles of Scilly Maritime Archaeology Society, 10 Tolver Place, Penzance TR18 2AD, UK.; 8 School of Geography, Earth and Environmental Sciences, University of Plymouth, Plymouth PL4 8AA, UK.; 9 Department of Environment and Geography, University of York, Heslington, York YO10 5NG, UK.; 10 Department of Earth Sciences, University of Hong Kong, James Lee Building, Pokfulam Road, Hong Kong, Hong Kong.; 11 Policy and Evidence, Historic England, Cannon Bridge House, 25 Dowgate Hill, London EC4R 2YA, UK.; 12 Institute for Biodiversity and Ecosystem Dynamics, University of Amsterdam, 1090 GE Amsterdam, Netherlands.; 13 School of History, Archaeology and Religion, Cardiff University, John Percival Building, Colum Drive, Cardiff CF10 3EU, UK.; 14 Geography, Royal Holloway, University of London, Egham TW20 0EX, UK.; 15 Department of Geography and Earth Sciences, Aberystwyth University, Aberystwyth SY23 3DB, UK.; 16 Geography, University of Exeter, Peter Lanyon Building, Treliever Road, Penryn TR10 9FE, UK.; 17 Cornwall and Isles of Scilly Historic Environment Record, Cornwall Council, Kresen Kernow, Little Vauxhall, Redruth, Cornwall TR15 1AS, UK.; 18 Colossus, Pilot’s Retreat, St Mary’s, Isles of Scilly TR21 0PB, UK.

## Abstract

Rising sea levels have been associated with human migration and behavioral shifts throughout prehistory, often with an emphasis on landscape submergence and consequent societal collapse. However, the assumption that future sea-level rise will drive similar adaptive responses is overly simplistic. While the change from land to sea represents a dramatic and permanent shift for preexisting human populations, the process of change is driven by a complex set of physical and cultural processes with long transitional phases of landscape and socioeconomic change. Here, we use reconstructions of prehistoric sea-level rise, paleogeographies, terrestrial landscape change, and human population dynamics to show how the gradual inundation of an island archipelago resulted in decidedly nonlinear landscape and cultural responses to rising sea levels. Interpretation of past and future responses to sea-level change requires a better understanding of local physical and societal contexts to assess plausible human response patterns in the future.

## Teaser

Past landscape change and human response to rising sea levels in island settings are shown to be nonlinear.

## INTRODUCTION

Global mean sea level is currently rising at 3.6 mm/year (*1*), and this rate of rise is unprecedented within recent millennia (*2*). However, the spatial distribution of sea-level rise is nonuniform across the globe (*3*). This means that some regions will experience rates of sea-level rise that are greater than the global average and are therefore particularly vulnerable to the associated hazards (*4*). Climate warming and persistent sea-level rise will cause increased extreme sea levels as a result of changes in water levels, tidal dynamics, wave climates, and storm surges (*5*). While future projected global and regional sea-level changes are becoming more tightly constrained, the cultural and behavioral responses of communities to these coastal hazards remain unpredictable. Estimates of future coastal flood risk to human populations (*6*, *7*) and projected migration patterns (*8*) are regularly based on analytically derived environmental thresholds and often disregard societal adaptation limits that can be driven by risk perception and culture (*9*). Catastrophic flooding events linked to prehistoric sea-level rise have been associated with rapid mass migration (*10*, *11*), yet this is a relatively simplistic view of human response to sea-level rise. Increasingly evident is that the impact of past sea-level rise on prehistoric coastal communities is not so straightforward (*12*). A pressing challenge for modern societies is to better understand the complex interactions between climate drivers, the consequent environmental changes, and human behavioral response. Here, we focus on past transient changes and responses that have occurred during a process of sea-level rise and coastal submergence.

Cultural transitions during the Holocene (past 11,700 years) have spread through societies as marked changes in technological advancement. However, these transitions have occurred within contexts of, and possibly related to, (sometimes rapid) climate and environmental change, including sea-level rise (*13*). Rates of global sea-level rise during the Early Holocene, leading up to the transition from Mesolithic hunter-gatherer societies to the farming-based practices of the Neolithic, were equal to or greater than modern rates due to the rapid disintegration of ice sheets over North America and Scandinavia (*14*). The migration of coastal communities displaced by this sea-level rise is a possible cause for the spread of the Neolithic transition throughout Europe (*13*, *15*). Rising sea levels may also have offered new opportunities for migration across northwest Europe via the expanding seaways (*16*, *17*). As well as opportunities, however, rising sea levels pose a range of threats (flooding, coastal erosion, land loss, habitat, and resource loss) to coastal communities. Within this context, insular societies, particularly island communities, are often most vulnerable to the effects of change due to issues of scale and isolation (*18*).

Here, we examine past human response to sea-level rise within a broader context of environmental change by using an isolated island group that has undergone fragmentation over the past c. 12,000 years to assess the nature of cultural transitions under pressure from rising seas. We calculate rates of sea-level rise for the Isles of Scilly in northwest Europe (hereafter referred to as Scilly) from a newly developed sea-level reconstruction (Materials and Methods), which allows us to assess rates of land loss and changes in areal extents of intertidal zones due to inundation. By combining new pollen and charcoal data from 17 sediment cores, dated using radiocarbon and optically stimulated luminescence (OSL), with reconstructed population dynamics from regional archeology, we show rapid landscape changes during a period of gradual sea-level rise, coincident with a period of major cultural change. These data suggest that prehistoric populations, in particular during the Bronze Age, adapted to, rather than abandoned, new island configurations.

## RESULTS AND DISCUSSION

### From island to archipelago

**Fig. 1:**
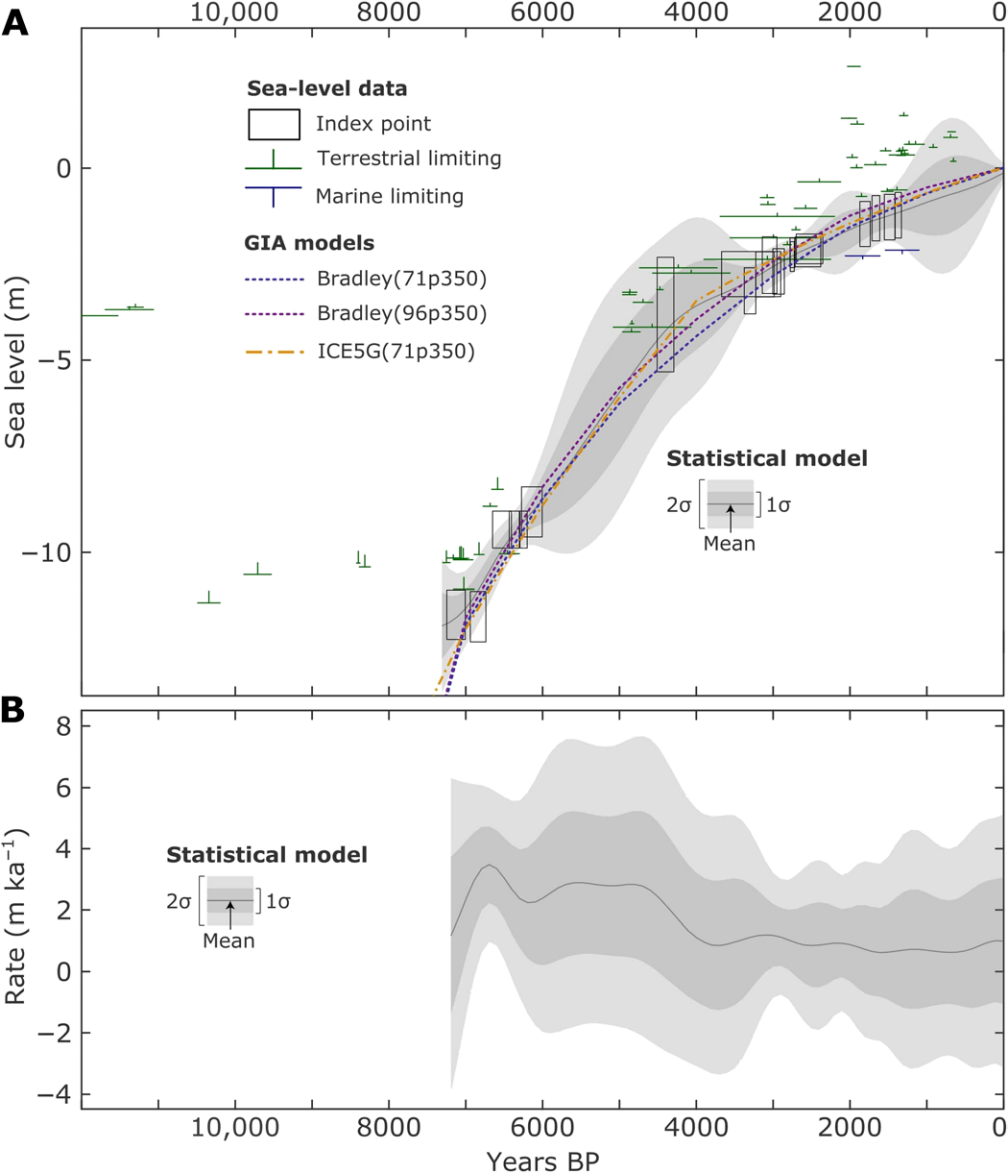
Holocene sea-level change. Reconstructed and modeled sea level and rates of sea-level change at Scilly. (**A**) Proxy sea-level data from dated sediments as precise data points (black boxes) with 2σ dating (horizontal) and reconstruction (vertical) uncertainties as well as limiting data points for sea-level maxima (green) and minima (blue) also with 2σ dating (horizontal) uncertainties. Gray shading is a statistical regression of the proxy data to provide a probabilistic sea-level envelope with mean (gray line), 1σ (dark gray), and 2σ (light gray) distributions. Colored dashed lines are relative sea-level outputs from a selection of the best-performing glacial isostatic adjustment models used in the study to extend the sea-level record beyond 7 ka. (**B**) Rates of sea-level change calculated from the statistical regression of the proxy data with mean (gray line), 1σ (dark gray), and 2σ (light gray) distributions. Years BP, years before present.

**Fig. 2:**
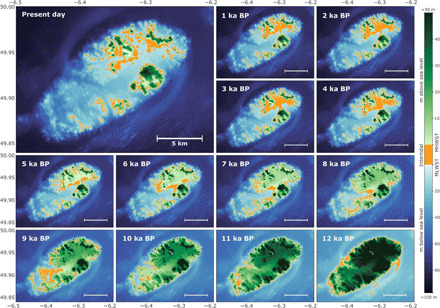
Holocene paleogeographies of Scilly. Changes in topography (green), bathymetry (blue), and intertidal area (yellow) modeled from the present-day values and extended back in time using relative sea- and land-level adjustments from the Bradley(71p350) glacial isostatic adjustment model and corrected for changes in tidal range.

Sea level around Scilly rose rapidly in the Early Holocene [defined as the period from 11.7 to 8.2 thousand years (ka)] (*19*) following the decay of the large Northern Hemisphere (Laurentian and Fennoscandian) ice sheets (*14*). Our sea-level reconstruction (Fig. 1) shows that sea level was still rising rapidly in the Mid-Holocene (8.2 to 4.2 ka) (*19*) at a rate of over 2 mm/year from around 7 ka [2.8 ± 1.4 (1σ) mm/year for 7 to 4.5 ka], gradually slowing to less than 1 mm/year after 4.5 ka [0.8 ± 1.6 (1σ) mm/year for 4 ka to present] for the remainder of the Holocene. This change in rate is due to the end of large scale ice-sheet melt and slowing of isostatic adjustment in Britain (*20*). The sea-level reconstruction relies on proxy-based observational data, but the sea-level history of Scilly can be extended back further by using relative sea-level outputs from best-performing glacial isostatic adjustment models (Materials and Methods). Modeled sea-level (Fig. 1A) and paleogeographies (Fig. 2) show that as late as 12 ka, Scilly was a single island, during a time when the island was intermittently visited, but lacked evidence of permanent settlement (*21*, *22*). As sea level rose, large intertidal zones developed by around 9 ka, and by 8 ka, the single original land mass had broken into a series of separate islands in the southwestern part of the modern archipelago. The modern island group remained a single land mass until the start of the Late Holocene (4.2 ka to present) (*19*), developing a very extensive intertidal zone between 5 and 4 ka, and only finally breaking into the separate islands that form the current configuration of islands and present-day tidal ranges during the past 1000 years.

**Fig. 3:**
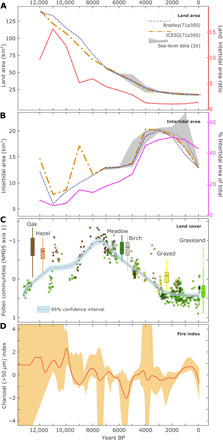
Environmental and landscape change. Landscape changes over the past 13,000 years. (**A**) Decline in total land area (defined as area in km^2^ above mean high water spring tides) and (**B**) change in area of the intertidal zone, determined from observational sea-level changes (gray shading) and relative sea-level outputs from glacial isostatic adjustment models (dashed lines). The changing ratio of land area to intertidal area (A) and the intertidal area as a percentage of the total area above mean low water spring tides (B) are also shown on secondary axes. (**C**) Vegetation cover index based on the first nMDS axis of the screened (intertidal and coastal samples removed) pollen data (filled circles) that has also been classified into community clusters (circle colors; fig. S1). (**D**) Fire index derived from counts of macro (>50 μm) charcoal from pollen samples from 17 of the sediment cores and monoliths from Scilly that were used to develop a composite charcoal curve.

The areal extent of the land and intertidal zones (Fig. 3, A and B) altered most rapidly in the Mid- to Late Holocene (despite the rapid sea-level rise over the Early Holocene), a result of the low gradients of the near-shore bathymetry allowing small changes in sea level to inundate relatively large areas of land. Terrestrial land area gradually reduced over the Holocene, concomitant with sea-level rise, with rates of land area loss slowing at c. 7 ka and again at 4 ka. However, the area of the intertidal zone increased gradually over the Early and Mid-Holocene and then rose very substantially between 5 and 4 ka, associated with the breakup of the single northwestern large island into five main distinct separate islands, physically joined only at low tide. The combination of gradual loss of available land area (Fig. 3A) and the fluctuating but overall increase in intertidal area (Fig. 3B) resulted in a very notable change in the character of the islands in terms of the proportion of intertidal area, as well as the further fragmentation and separation of the islands. The relatively modest and gradual sea-level rise in the Late Holocene resulted in major landscape change. Given the evidence for repeated human settlement on Scilly by the Mid- and Late Holocene (*17*, *21*, *22*), the changing environment would have led to substantial alterations in the practical constraints and opportunities for the local human population, as well as driving very different societal and cultural perceptions of the landscape.

### Land availability and resources

Overall potential land availability became increasingly limited as sea levels rose. The increase in areal extent of the intertidal zone would have created opportunities through an increase in accessible intertidal resources and also limitations in communication and ease of movement between the fragmenting islands. A critical question in such transitions is how this millennial-scale change would have been manifested on human time scales. Would this have been perceptible to individuals and communities and in oral tradition? There is lithic (*16*, *21*) and palynological evidence (*22*, *23*) for seasonal occupation on Scilly during the Late Mesolithic, with more persistent settlement during the Middle Neolithic suggested from land clearance, ceramics, and agricultural practice (*22*, *23*). During these periods, the average loss in land area reduced from ~15.5 km^2^ per millennium in the Early Holocene to ~12.5 km^2^ per millennium during the Mid-Holocene. Despite the rate of loss slowing during the Mid-Holocene, the loss of land relative to the land area at the time was substantial; ~36% of the available land area at that time was lost to submergence between 8 and 4 ka. This rapid land loss was accompanied by expansion in intertidal areal extent at rates of around 1 to 5 km^2^ per millennium, representing an increase from around 5 to 28% of these environments. These millennial-scale changes would have been observable on human time scales. A single 70-year lifespan (the modal lifespan for hunter-gatherers living today) (*24*) would have witnessed a decrease of more than 700,000 m^2^ in land area and an increase of over 350,000 m^2^ in intertidal area. These changes would be substantially greater in the personal experience of the oldest individuals and in oral history, which on a conservative basis would extend over perhaps a few hundred years. Furthermore, three additional factors mean that these changes would be much more consequential to the individuals and populations present around 4.5 ka (Late Neolithic-Chalcolithic transition) than these numbers imply.

First, the rate of landscape change is not constant. In particular, some of the most rapid changes occur between 5 and 4 ka. Rates of land loss are similar to the Mid-Holocene average of ~12.5 km^2^ per millennium, but the rate of increase in area of intertidal zone accelerates markedly during this time to over 5 km^2^ per millennium, more than three times the average rate of gain throughout the Mid-Holocene. Second, the island area was already considerably reduced by 5 ka such that the proportional change in the land surface from 5 to 4 ka (−36%) was much more extreme than the absolute change in area (−10 km^2^) and relatively greater than at any other time before or after this. Likewise, the proportion of the intertidal zone relative to land area changed markedly from 28% to almost 50% by 4 ka. Third, the distribution of landscape change was not evenly spread geographically. The more gradual changes in the Early and Mid-Holocene occurred almost entirely around the coastal fringes and in the western sector with the development of intertidal zones. The configuration of the islets in the western sector largely remained unchanged from the Mid-Holocene to present day. The very large shift in land and intertidal area between 5 and 4 ka occurred primarily in the northeastern sector and was accompanied by the physical separation of five main islands and the development of a vast new intertidal area between these (Fig. 2).

From the perspective of individuals and societies inhabiting the islands between 5 and 4 ka, the shift in landscape would have been remarkable. Every 10 years, ~100,000 m^2^ (or ~0.36%) of land was lost to rising sea level and over 50,000 m^2^ of new intertidal area was created, almost all within the northeastern sector between the three main islands. The lateral rate of land inundation is estimated at 1 to 3 m/year between these islands during the millennium between 5 and 4 ka. This lateral inundation and the associated erosion of the coastline, perhaps more so than the changes in available land area, would be observable over a human lifespan and certainly within oral traditions of local populations. These high rates of landscape change occurred within a rapidly diminishing rate of relative sea-level rise [i.e., from 2.8 ± 2.3 (1σ) mm/year at 5 ka to 1.1 ± 2.1 (1σ) mm/year at 4 ka]. Despite this relatively gradual sea-level rise, people were living through rapid landscape change and encroachment of the sea; a shrinking land mass and increasing intertidal zone would have been a constant backdrop. In the millennium between 5 and 4 ka, the largest remaining island became fragmented, ~36% of the land was lost and an extensive intertidal zone had been created, severing the link between communities at high tide.

It was not only the extent of land and intertidal zone that was altering. Variations in vegetation structure (Fig. 3C) reflect the combined effects of sea-level rise and land use but do not suggest a sequence of change greatly different to that on the mainland (*25*), with the exception of the local variations in intertidal habitats. Woodland cover had developed in the Early Holocene following climatic amelioration and vegetation succession from postglacial grasslands (Fig. 3C). Flint microliths found on Scilly from the Late Mesolithic (*16*, *21*) suggest that seasonal visitors to the archipelago (possibly from present-day France or Belgium) (*12*, *16*) were likely exploiting local resources through hunting, fishing, and gathering at this time. Climax vegetation biomes (oak woodlands) were achieved by the Mid-Holocene, although evidence of oak-dominated communities abruptly ends at c. 7 ka (Fig. 3C). Occasional peaks in charcoal records (Fig. 3D) suggest that reduction in woodland cover during the Early and Mid-Holocene may have been accelerated by anthropogenic burning, potentially indicating a marked change in land use at 7 ka.

**Fig. 4:**
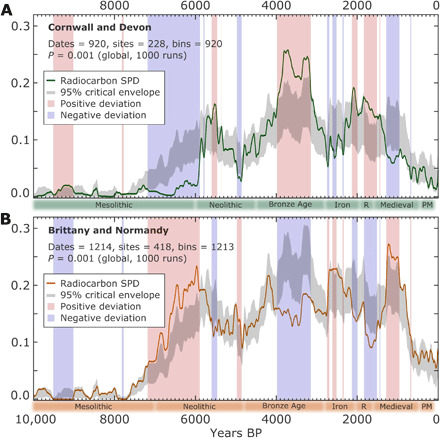
Regional population demographics. Population demography estimated using SPDs of archeological radiocarbon dates. Radiocarbon SPD curves for Devon and Cornwall (**A**) and Brittany and Normandy (**B**) shown as solid colored curves. Iterations of random permutations (*n* = 1000) of the global (Devon and Cornwall plus Brittany and Normandy) radiocarbon dataset are used to develop a theoretical global trend with 95% critical thresholds (gray shading). Departures of local SPD curves above (red shading) and below (blue shading) this theoretical global model are shown to denote regionally specific and significant population trends.

Reduction in woodland cover predates population expansion on the adjacent mainland (Cornwall and Devon) by 1000 years (Fig. 4A) yet is concomitant with the onset of the continental Neolithic in northwest France at 7 ka (Fig. 4B). Late Mesolithic activity on Scilly may therefore provide evidence for the importance of sea-faring in the spread of new practices and people throughout northwest Europe (*16*). As sea levels rose, salt marsh and sedge meadows developed in the low-lying areas, and by the time of the rapid coastal changes between 5 and 4 ka, land was being used for grazing and small-scale cultivation (Fig. 3C) (*22*, *23*, *26*, *27*). The salt marsh and fringing freshwater marsh that developed would have created an expanded resource for foraging, and as the extent of intertidal zone developed, it would have been a substantial resource for foraging, fishing, and hunting. The consequences of sea-level rise for people were thus not simply negative (a reduction in available land) but would have created the opportunity for new modes of subsistence and communication.

### Cultural and societal change

The rapid changes in areal extent of land and intertidal zones between 5 and 4 ka span the end of the Neolithic and the beginning of the Bronze Age in the British Isles around 4.2 ka (*28*, *29*). It might be assumed that the dwindling land area and fragmentation of the islands would lead to a reduction in human presence or even abandonment [e.g., due to flooding and coastal salinization (*11*)]. The evidence from the archeological record does not support this and alternatively suggests a substantial acceleration of activity. Repeated settlement on Scilly during the Middle Neolithic is evident from land disturbance and archaeological features and may be further inferred during the Late Neolithic with the arrival of grazing stock (*17*, *22*, *23*), yet the increase in archeological evidence from the Early Bronze Age is startling (fig. S3). The abundance of material culture in the form of lithics (worked flint, chert, and quartz) and ceramics (pottery sherds and vessels) is evident (*22*). More notable though is the widespread flourishing of Early Bronze Age monumentality on Scilly. With over 600 recorded cairns, standing stones, entrance graves, and other monuments, in addition to Middle and Late Bronze Age settlement structures and field systems, the archeological resource is richer on Scilly in the Bronze Age than during any other period (fig. S3) and represents the highest density of monumentality in southwest Britain (*22*, *30*). Although precise dating of these monuments has been difficult, reanalysis of pottery coupled with accelerator mass spectrometry (AMS) radiocarbon dates identifies the main period of use of entrance graves to the Early Bronze Age as opposed to the Neolithic (*31*, *32*). Any dated evidence that might suggest earlier entrance grave construction (e.g., to the Late Neolithic) remains absent for the time being.

Currently outstanding is whether this remarkable increase in Early Bronze Age activity on Scilly is concurrent with the onset of the Bronze Age on mainland Britain. Population demographics for southwest Britain and northwest France, inferred using radiocarbon summed probability distributions (SPDs) (Fig. 4), show that substantial Bronze Age expansion in southwest Britain occurs at 4 ka or slightly earlier, at a time of demographic decline in northwest France. Whatever the precise chronology of construction of this extraordinarily dense prehistoric monumental landscape, its development shows that human activity on the islands did not decline but intensified in the closing stages of the fragmentation of the mainland area of Scilly. It is unclear whether monument building was accompanied by permanent settlement, but it seems unlikely that the effort of monumentalization could have been maintained without some permanent presence. The lack of direct evidence may simply be a result of poor preservation or submergence of structures. The relict field systems that can be observed in the present-day intertidal zones across Scilly are likely to be Bronze Age based on their elevations and typology, indicating settled populations at the time (*23*).

Was the rapid expansion in Early Bronze Age activity on Scilly a result of cultural and societal shifts driven by changes in the character and extent of the available land described above? While this cannot be definitively answered, the synchronicity between the timing of the largest changes in coastal configuration and the increase in monumentality suggests that the two are linked. An expansion in monument building is also apparent in Cornwall on mainland Britain after 4 ka (*30*). As is often the case with prehistoric monuments, the use and reasons for construction of the entrance graves and other monuments remain unknown, with existing explanations ranging from tombs and communal shrines to territorial markers or navigation aids for seafarers. Reasoning behind the locale of monuments is also debated. Locations may simply be focused proximally to construction resources or in areas of low agricultural value, or they may have cultural importance, potentially relating to the immediate environment. The idea of connections between seafaring and the location of entrance graves is disputed (*23*, *31*, *32*) but should not be dismissed given Scilly’s situation within maritime networks (*16*, *17*). Alternatively, could this intensity of monument building be in some way reactive to the large-scale coastal landscape changes that occurred across the Late Neolithic–Chalcolithic–Early Bronze Age transition?

While a contemporary logical response to reduction in land area and resource availability might be to retreat and seek a new place to live, on Scilly the drive to adapt was greater and the response may have been cultural rather than physical. Responses to external change are embedded within social groups, and adaptive responses are mutable and subjective (*9*). A sense of ownership and belonging is powerful, especially where change is experienced throughout multiple lifetimes and is, therefore, accepted as an inevitable part of the environment. Prehistoric insular communities may be particularly resilient on the basis of strong social and environmental ties: A diversity of economic resources (such as food from the intertidal and coastal zone around Scilly) may supplement resources derived from the terrestrial ecosystem. Elsewhere along the Atlantic margin, the development of marine resource availability is a clear asset to prehistoric coastal communities (*12*). Present-day resistance to climate-driven relocation is evident within modern communities in low-lying islands (*33*, *34*), where centering societal and cultural perspectives at the heart of climate mobility discourse is paramount (*35*). Here, we present rare evidence that this focus on societal and cultural practices during sea-level transitions has likely been important for thousands of years.

The physical complexities of coastal landscape response to sea-level rise are gradually being built into sea-level rise impact assessments (*36*). That said, there remains an insufficient appreciation of the ways in which bathymetry, topography, sediment dynamics, and tidal regimes modify the physical manifestation of gradual sea-level rise, especially in island settings such as the Isles of Scilly. The nonlinear changes in the spatial configuration of land and intertidal areas that arise from these factors need to be considered to elucidate patterns of past coastal changes and inform projections of future sea-level rise. In particular, given the regional disparity in rates of ongoing and future sea-level rise (*37*), we demonstrate that large-scale coastal reorganization is not necessarily restricted to entities of space and time undergoing the highest rates of relative sea-level rise. It is apparent that rapid coastal change can occur within regimes of relatively small and gradual sea-level change. Likewise, human response patterns to these physical changes are equally nonlinear and may be far less predictable than simplistic models of inundation and reactive migration imply. As can be seen today across island nations, cultural practices define the adaptive response of coastal communities, which can result in polarized agendas, such as the planned relocation programs in Fiji (*38*) versus the climate-migration resistance seen in Tuvalu (*34*). It is perhaps unlikely that coastal reorganization in coming centuries will lead to new resource availability on scales capable of supporting entire communities, as found on Scilly for past millennia. More certain though is that societal and cultural perspectives from coastal populations will be critical for developing holistic and successful adaptive responses to future climate change.

## MATERIALS AND METHODS

### Experimental design

We undertook field studies to collect sedimentary records of past sea level and landscape changes. Recovered sediment cores were dated using OSL and radiocarbon techniques and underwent age-depth modeling. Paleoenvironmental reconstructions were based on palynological analyses (including charcoal), phytosociological classifications, and nonmetric multidimensional scaling (nMDS). Sea-level reconstructions were based on foraminiferal analyses and a combined transfer-function/indicative meaning–based approach. Bayesian inference of sea-level trends from observational data was modeled using errors-in-variables integrated Gaussian process. Sea-level trends were extended beyond observational data using glacial-isostatic adjustment (GIA) modeling. Paleogeographies were developed by combining a model of present-day bathymetry and topography with GIA model outputs and a new paleotidal range model. Archeological indices were developed using SPDs of regional archeological radiocarbon dates and by using a database of local, age-inferred archeological evidence.

#### Surveys and core collections

Surveys and core collections took place across Scilly in 2009 and 2010 during low spring tides. An audit of intertidal peat deposits that were recorded by (i) English Heritage in the Intertidal and Coastal Peat Database, (ii) the Historic Environment Service in the Rapid Coastal Zone Assessment for the Isles of Scilly, and (iii) the Cornwall Archeological Unit in The Early Environment of Scilly (*27*) was carried out and digitized by Cardiff University (*23*). The audit guided field surveys of extant deposits, which were surveyed using a Trimble 4700 RTK GPS base and rover system and converted to the British National Grid coordinate system using the Ordnance Survey (OS) National Grid Transformation OSTN02. Elevation was converted to Ordnance Datum St Mary’s using OS National Geoid Model OSGM02. Baseline postprocessing used local reference stations from the OS National Survey GPS Network to reduce GPS position errors. Mean horizontal and vertical positional uncertainties across all surveys were ±0.04 and ±0.10 m, respectively. Sediment cores for paleoenvironmental analyses were collected as hand-cut monoliths from 15 locations exhibiting thick peat sequences (table S1).

The Cornwall and Isles of Scilly Maritime Archeology Society (CISMAS) carried out a submarine geophysical survey on board the vessel Tiburon of DiveScilly to map subtidal peat deposits around the islands using a type C-Max CM2 side-scan sonar with Garmin 76C EGNOS–enabled GPS for positioning (*23*). An area of 1.35 km^2^ across seven search areas was surveyed in 2009, revealing subtidal peat deposits in two search areas. The thickness of the deposits was measured with a SyQwest Stratabox subbottom profiler, and search areas were ground-truthed during 29 dives by CISMAS divers. Sediment cores were collected at 13 sites using percussion coring and hand-cut monoliths and geolocated using a Garmin 76C EGNOS–enabled GPS with Chart Depth and then estimated from Admiralty Chart number 0883 (table S1).

#### Chronologies

Chronologies were developed for the collected cores and monoliths using radiocarbon and OSL dating techniques. Samples of plant and animal macrofossils, wood, charcoal, and bulk organic sediment were sent for radiocarbon dating at either the Oxford Radiocarbon Accelerator Unit (OxA), University of Oxford, or the Scottish Universities Environmental Research Centre (SUERC), University of Glasgow. Sample pretreatment and AMS measurements followed OxA and SUERC laboratory procedures. Where identifiable macrofossils were unavailable for dating, humin and humic fractions of bulk organic sediments were dated following pretreatment and age determinations were calculated using weighted means (*23*). Of the 70 samples sent for dating, 78 age determinations were returned from the two facilities; replicate determinations were obtained from the humin and humic fractions of organic bulk sediment and 16 samples failed during pretreatment due to low carbon yields. Age determinations were catalogued with relevant sample metadata (stratigraphy, depth, unit thickness, positioning, elevation, and error estimation) in dataset S1.

For OSL dating, samples were taken from sediment monoliths under subdued red-lighting conditions. The light-exposed surface material of the sediment monoliths was removed and used for assessment of the dose rate (Gy/ka), based on measurements of finely ground material made using Daybreak detectors for thick source alpha counting and a Risø GM-25-5 beta counter. The units subsampled for OSL dating were all within 20 cm of the uppermost surface of the 30-cm-long monolith tin and were bracketed by different stratigraphic units. The gamma dose rate to the sample was therefore calculated according to the principles outlined in Appendix H of Aitken (1985) (*39*), using the multilayer gamma model of I. Bailiff and S. Barnett (University of Durham). The non–light-exposed sediments within the monolith were used to determine the equivalent dose (*D*_e_, Gy). Coarse-grained (i.e., sand-sized) quartz was prepared for dating, using treatment with a 10% (v/v) dilution of 37% hydrochloric acid (HCl) to remove carbonates, followed by treatment with 20 volumes of hydrogen peroxide (H_2_O_2_) to remove organics, before sieving to narrow (~20- to 30-μm interval) grain-size ranges and density separation using sodium polytungstate to separate the quartz from other minerals. The quartz-rich fraction was then treated with hydrofluoric acid (HF; 40% for 45 min) to remove the alpha-irradiated outer surface of the quartz grains and to dissolve any feldspars present, followed by treatment with concentrated (37%) HCl for 45 min and finally resieved on drying as a further purification step.

Measurement sequences for quartz OSL dating were applied to 24 aliquots of each sample, which were screened using signal intensity, background signal levels, recuperation, recycling, and OSL infrared depletion ratio criteria (see English Heritage RRS 2013-2 for details) (*39*). The minimum number of aliquots passing screening criteria for any given sample was 18/24 (table S2); the few aliquots that failed these screening criteria tended to do so on the basis of recycling ratios exceeding ±10% of unity. Values of *D*_e_ were calculated as weighted means of screened aliquots with root mean square error (RMSE) SEs, with the exception of one sample (161/LPPM1-1), where the simple arithmetic mean of the *D*_e_ values and SD was used to reflect the broad distribution and hence relatively large uncertainty in the *D*_e_ value (table S2). The dose rate, *D*_e_ values, and final ages determined for each sample are shown in table S1, expressed in years relative to a datum of 1950 CE to enable direct comparison with calibrated radiocarbon ages. Analysis of two modern analog intertidal surface samples (161/LPTR1-M and 161/LPT3-M) gave results equivalent to burial for 3 ± 2 years for both samples, suggesting that incomplete bleaching is not a problem for the intertidal samples in this OSL dating study.

Bayesian age-depth modeling was used to provide chronologies for sediment cores and monoliths that contained multiple dates from radiocarbon and OSL dating (*23*). Age-depth models were built using OxCal v4.1 (*40*) using the IntCal13 calibration curve (*41*) with the *P*-sequence function and the deposition rate prior defined as log_10(*k*/*k*_0), where *k*_0 = 1, allowing *k* to take any value between 0.01 and 100. Highest posterior density intervals throughout the sediment cores are reported in calibrated years before present (1950 CE) as modeled mean and 2σ ranges (dataset S2).

#### Paleoenvironmental reconstructions

Paleoenvironmental reconstructions were based on pollen of the sediment cores and monoliths, which were subsampled at 1-cm resolution (*23*). Sediment subsamples (1 cm^3^) were combined with *Lycopodium clavatum* L. as an exotic spike in 10% HCl (4 ml) to provide concentration data. Samples were then digested in 10% NaOH (40 ml) before sieving (15- to 106-μm fraction), treated with 10% HF (4 ml), washed with 10% HCl, and underwent acetolysis before being stored in glycerol for counting. Pollen counts continued until 300 land-derived grains had been identified in each sample. Charcoal counts on two size fractions (>50 and <50 μm) were carried out simultaneously.

Pollen assemblages were assigned to clusters using Ward’s hierarchical agglomerative clustering using the “rioja” software package (*42*) in R (*43*). This unsupervised data-driven approach was used to assign pollen samples to cluster groups based on the similarity of their taxa assemblages. After assigning pollen samples to clusters statistically, a phytosociological classification approach was used to identify the frequent and abundant taxa within each group based on the number of occurrences of the taxon (the frequency), the average percentage, median, and interquartile range (*26*). An individual taxon’s frequency is determined by calculating its number of occurrences divided by the number of samples in the cluster and assigning one of five frequency classes based on cutoff values between each group. If a taxon appears in 81 to 100% of all samples in the cluster group, it is assigned the highest frequency class. nMDS was applied to the data using the “vegan” software package (*44*) in R (*43*) as a complementary method to summarize major variation in the dataset (fig. S1). The stress of the nMDS was used as an indicator of the quality of the fit of the ordination.

The results of the phytosociological classification (tables S3 to S6) were used to identify samples that had a greater influence of coastal processes. This was deemed necessary to differentiate pollen assemblages that have a greater forcing through human influence from those driven by coastal change (*26*). Clusters that included key halophytes in high frequencies (e.g., Chenopodiaceae) were screened from the dataset used to develop the land cover index for Scilly. The remaining samples were first orientated in time (*x*-axis age values based on age-depth modeling results of the sediment cores) and space (*y*-axis values based on the primary nMDS ordination axis) before regression analysis to determine a trend through the data. The regression used generalized additive models to estimate smooth, nonparametric link functions between the two variables, fit to the data using penalized regression splines in the “mgcv” software package (*45*) in R (*43*) with uncertainties determined as the variance of the SEs of the estimated trend in time.

To examine biomass burning across Scilly during the Holocene, charcoal records were analyzed using the “paleofire” (v. 1.2.3.) software package (*46*) in R (*43*). Seventeen charcoal records from the analyzed sediment cores and monoliths (table S1) were included in the analysis to create a composite charcoal curve. To facilitate intersite comparison, the 17 records were pretreated using the established protocol for transforming and standardizing individual records: (i) transforming noninflux data (e.g., particle concentration values in cm^−3^) to influx values (particle cm^−2^ year^−1^), (ii) homogenizing the variance using a Box-Cox transformation, (iii) rescaling the values using a min-max transformation to allow comparisons among sites, and (iv) rescaling the values to *z* scores using a base period of 200 years. Sites were smoothed with a 300-year half-width smoothing window and a bootstrap of 100 years (*46*).

#### Sea level

Sea level was reconstructed using assemblages of intertidal foraminifera from the fossil sediments as precise sea-level indicators, and subsampling was concentrated along sediment cores and monolith sections that exhibited high concentrations of Chenopodiaceae pollen. Known volumes of sediment (1 to 5 cm^3^) were washed through 63- and 500-μm mesh sieves, wet-split into equal aliquots, and counted until >100 tests were identified or until the entire sample had been counted (*23*). Paleomarsh surface elevations were estimated from samples with count totals of >50 tests by applying a two-component weighted average partial least squares transfer function model with inverse deshrinking to predict sample elevations from relative abundances of foraminifera (*23*). Paleomarsh surface elevation predictions from the fossil assemblages and their bootstrapped root mean squared prediction errors (RMSEPs) were corrected for tidal range differences between the training set location (Erme Estuary, South Devon) and Scilly (St Mary’s tide gauge) before being converted to estimates of sea level, following: S = H – I, where the position of former sea level (S) is the elevation of the sample relative to MSL (H) minus the indicative meaning (I) of the sample, in this case, the paleomarsh surface elevation result from the transfer function (dataset S2).

The transfer function approach provided 14 precise estimates of former sea level with uncertainties calculated as 2σ RMSEs of surveying, sampling, and transfer function errors. These samples all paired with precise dating (radiocarbon or OSL) constraints, which provided corresponding 2σ age uncertainties. The remaining age determinations were used to develop a further 56 sea-level index points (SLIPs), 8 of which were precise and the remainder were marine- or terrestrial-limiting. Indicative meanings for these SLIPs were based on lithologies and paleoenvironmental analyses (dataset S1), and the corresponding reference water levels and indicative ranges were based on those used for the sea-level database of Britain and Ireland (*20*). The 70 new index points developed in this study (dataset S1) were combined with the 20 existing limiting data points for Scilly (*26*) from the British and Irish sea-level database (*20*) to produce a new Holocene sea-level database and sea-level curve for Scilly.

Temporal trends and uncertainties in relative sea level were estimated for the Scilly region by applying an error-in-variables integrated Gaussian process (EIV-IGP) model (*47*) to the combined relative sea-level dataset. This method incorporates sample-specific vertical and temporal uncertainty and the uneven distribution of data points through time. Uncertainties reported in the text from the EIV-IGP model are a mean with 95% credible interval.

#### Tidal range

Tidal range changes through the Holocene were simulated for Scilly using a paleotidal model of the northwest European shelf seas (*48*). The Regional Ocean Modeling System was used to develop a three-dimensional tidal model, configured with latitude/longitude grid spacing of 1 to 2 km. Tidal elevation amplitudes were output at 1-ka intervals from 21 ka to present day, with boundary forcing for the paleotidal runs derived from a global paleotidal model (*49*). Effects of vertical land motion were estimated using 1-ka time slices from a GIA model for the United Kingdom (*50*) in combination with present-day bathymetry. Simulated tidal range changes were used to correct the indicative ranges of the sea-level indicators used in this study (dataset S1) and to account for tidal amplitude changes when calculating the area of the intertidal zone through time.

#### Glacial isostatic adjustment

Glacial isostatic adjustment models were used to derive relative sea-level predictions for the study region and extend sea-level histories beyond the extent of the observational data. The glacial isostatic adjustment model used builds upon Bradley *et al*. (*50*). The model was run at a spherical harmonic degree of 512° to provide ~35-km resolution across the study region and considers two different global ice-sheet models, Bradley2017 (*20*) and ICE5G (*51*), which prescribed the evolution of major ice sheets from ~122 ka to present day. Sensitivities to the choices of Earth rheology were investigated by generating relative sea-level predictions for a range of Earth model parameters with χ^2^ misfits calculated by

$$\chi^{2}=\frac{1}{\left(N-1\right)}\overset{N}{\underset{i=1}{\Sigma }}{\left(\frac{\left({\text{RSL}}_{i}^{p}-{\text{RSL}}_{i}^{\text{obs}}\right)}{\sigma_{i}}\right)}^{2}$$

where *N* is total number of precise SLIPs; ${\text{RSL}}_{i}^{p}$ and ${\text{RSL}}_{i}^{\text{obs}}$are the predicted and observed relative sea-level data defined at given latitude, longitude, and time; and σ*_i_* is 2 sigma errors on the observed SLIP (dataset S1). For a lithosphere thickness of 71 km, the minimum and maximum ranges of upper and lower mantle viscosities are 0.1 × 10^21^ to 1 × 10^21^ Pa and 1 × 10^21^ to 10 × 10^21^ Pa, respectively (54 models in total). For a lithosphere thickness of 96 km, the ranges are the same, although only 48 models were used to produce χ^2^ misfit contour plots (fig. S2). Earth models with the lowest χ^2^ misfit for each global ice-sheet reconstruction were selected for use in the study. This produced models with a lithosphere thickness of 71 km and upper and lower mantle viscosities of 0.3 × 10^21^ Pa and 50 × 10^21^ Pa, respectively, for both Bradley2017 [Bradley(71p350)] and ICE5G [ICE5G(71p350)] ice-sheet histories. An additional high-performing model with a 96-km lithosphere thickness and 0.5 × 10^21^ Pa and 30 × 10^21^ Pa upper and lower mantle viscosities using the Bradley2017 ice-sheet history was also selected for comparison.

#### Paleogeographies

Paleogeographies for Scilly were developed using three sea-level histories: (i) the newly developed probabilistic relative sea-level curve (the 50% confidence interval) for 7.5 to 0 ka, (ii) the relative sea-level output for the Bradley(71p350) glacial isostatic adjustment model, and (iii) the relative sea-level output for the ICE5G(71p350) glacial isostatic adjustment model. Bathymetry data for the Isles of Scilly and surrounding waters were downloaded from the Channel Coastal Observatory (www.channelcoast.org; accessed June 2018). Separate ascii files of Lidar data, collected between 18 March 2014 and 18 June 2015, were collated and gridded in ArcGIS, with a grid resolution of 30-m offshore, increasing in resolution to 1.5 m in the intertidal zone and on land. Bathymetry and Lidar data were both referenced to present-day MSL. The relative sea-level histories were combined with the present-day bathymetric grid and corrected for paleotidal range changes by extracting the amplitudes of the M2 and S2 tidal constituents at 1-ka intervals using the paleotidal model outputs developed in this study. The paleogeographies and paleotidal ranges were then used to calculate land- and intertidal-area changes for the three relative sea-level histories through the Holocene at 1 ka intervals.

#### Archeological indices

Archeological indices were developed using two different methods. Time series of population demographic change were inferred for southwest Britain and northwest France using regional compilations of archeological radiocarbon dates to produce SPDs (*52*). This use of radiocarbon dates as a proxy for population has been extensively discussed, with considerable methodological response to concerns that the preserved signal might more often be dominated by biases in modern archaeological investigation intensity rather than by changing intensity of past human activity. Equally reassuringly, the SPD for southwest England used here preserves a similar signal to other parts of southern England and exhibits good congruence with evidence for likely anthropogenic landscape impacts provided by aggregated pollen records (*52*). The data compilations comprise 1410 archeological radiocarbon dates from Devon and Cornwall and 1424 dates from Brittany and Normandy, collated from over 150 databases and published resources (dataset S3; see “Data and materials availability” for database citations). Dates with analytical (e.g., low carbon yields), sampling (e.g., evidence of contamination), and provenance (e.g., environmental associations rather than archeological) issues were screened from both compilations, which resulted in final dataset sizes of *n* = 920 (Devon and Cornwall) and *n* = 1214 (Brittany and Normandy). The “rcarbon” software package (*53*) in R (*43*) was used to calibrate the remaining dates with the IntCal13 calibration curve (*40*) and then cluster dates from same sites that occurred within 50 year bins (i.e., similar aged dates) to avoid overrepresenting disproportionately sampled phases or archeological sites in the resulting radiocarbon SPD curves. Radiocarbon distributions were summed separately for the two regions without normalizing postcalibrated dates (*52*). A significance test of conditional (fixed dates but with randomized attribution of each date to one of the two regions), random permutations (*n* = 1000) was applied across the combined regions to develop a 95% confidence interval of “expected” radiocarbon SPDs. Local departures from this confidence interval were considered significant when the observed regional SPD curves occurred outside of this distribution of global permutations.

A further secondary archeological index was developed to provide an approximation of population variability on Scilly based on time-conditional densities of archeological evidence available from the landscape (fig. S3). A dataset (*n* = 2411) of recorded archeological monuments from Scilly (dataset S3) was obtained from the Cornwall and Scilly Historic Environment Record (heritagegateway.org.uk). Dataset metrics included classifiers (e.g., identifiers and locators), monument types (e.g., structures, settlements, cairns, entrance graves, boundary and field markers, findspots, middens, and wrecks), and inferred relative ages. The relative ages associated monuments with known (e.g., Bronze Age), or “windows” (e.g., Medieval to Modern) of known, archeological epochs, which were subsequently used to assign calendar dates to the monuments, based on established archeological period timings for southwest Britain (*22*). The index was developed using a probabilistic approach by assigning the likelihood of a monument occurring within its temporal range to 1 and summing probabilities of occurrence for all monuments in 200-year bins through the Holocene (fig. S3). This approach produces an aoristic sum, which is based on the age-certainty of each monument and downweights monuments with temporal uncertainty (i.e., occurring within large windows of age-inferred archeological periods). Data points of unknown relative ages were screened from the dataset before analysis.

## Supplementary Material

http://advances.sciencemag.org/cgi/content/full/6/45/eabb6376/DC1

Dataset S1

Dataset S2

Dataset S3

Adobe PDF - abb6376_SM.pdf

Nonlinear landscape and cultural response to sea-level rise

## SUPPLEMENTARY MATERIALS

Supplementary material for this article is available at http://advances.sciencemag.org/cgi/content/full/6/45/eabb6376/DC1
