# Supplementary material for: Nonlinear landscape and cultural response to sea-level rise
Source: Sci Adv. 2020 Nov 4;6(45):eabb6376. doi: 10.1126/sciadv.abb6376 (PMC7673675; doi:10.1126/sciadv.abb6376)
Supplement: http://advances.sciencemag.org/cgi/content/full/6/45/eabb6376/DC1 [file supp_6_45_eabb6376__index.html]

Science Advances | Science AdvancesAAASSearchScience AdvancesMenu

## Supplementary Materials

# Nonlinear landscape and cultural response to sea-level rise

Robert L. Barnett, Dan J. Charman, Charles Johns, Sophie L. Ward, Andrew Bevan, Sarah L. Bradley, Kevin Camidge, Ralph M. Fyfe, W. Roland Gehrels, Maria J. Gehrels, Jackie Hatton, Nicole S. Khan, Peter Marshall, S. Yoshi Maezumi, Steve Mills, Jacqui Mulville, Marta Perez, Helen M. Roberts, James D. Scourse, Francis Shepherd, Todd Stevens

Download Supplement

**The PDF file includes:**

- Figs. S1 to S3
- Tables S1 to S6
- Legends for datasets S1 to S3
- List of Radiocarbon Resources

**Other Supplementary Material for this manuscript includes the following:**

- Dataset S1
- Dataset S2
- Dataset S3

**Files in this Data Supplement:**

- Adobe PDF - abb6376\_SM.pdf
