# Supplementary material for: Nonlinear landscape and cultural response to sea-level rise
Source: Sci Adv. 2020 Nov 4;6(45):eabb6376. doi: 10.1126/sciadv.abb6376 (PMC7673675; doi:10.1126/sciadv.abb6376)
Supplement: http://advances.sciencemag.org/cgi/content/full/6/45/eabb6376/DC1 [file supp_6_45_eabb6376__1.pdf]

Science Advances
AAAS

advances.sciencemag.org/cgi/content/full/6/45/eabb6376/DC1

Supplementary Materials for

Nonlinear landscape and cultural response to sea-level rise

Robert L. Barnett*, Dan J. Charman, Charles Johns, Sophie L. Ward, Andrew Bevan, Sarah L. Bradley, Kevin Camidge, Ralph M. Fyfe, W. Roland Gehrels, Maria J. Gehrels, Jackie Hatton, Nicole S. Khan, Peter Marshall, S. Yoshi Maezumi, Steve Mills, Jacqui Mulville, Marta Perez, Helen M. Roberts, James D. Scourse, Francis Shepherd, Todd Stevens

*Corresponding author. Email: r.barnett@exeter.ac.uk

Published 4 November 2020, *Sci. Adv.* **6**, eabb6376 (2020)
DOI: 10.1126/sciadv.abb6376

The PDF file includes:

Figs. S1 to S3
Tables S1 to S6
Legends for datasets S1 to S3
List of Radiocarbon Resources

Other Supplementary Material for this manuscript includes the following:

(available at advances.sciencemag.org/cgi/content/full/6/45/eabb6376/DC1)

Datasets S1 to S3

Supplementary Figures

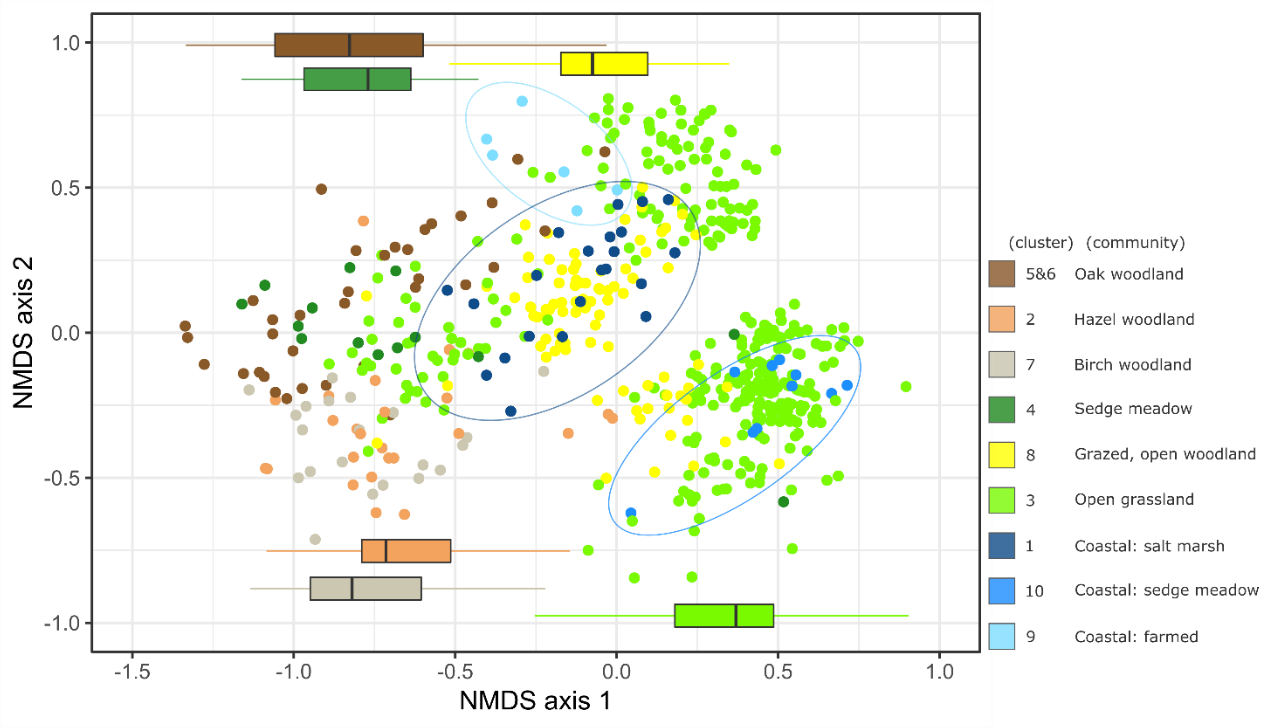

Fig. S1. Ordination plot of pollen sample data. This plot shows sample ordinations (dots) of the first two axes following a non-metric multidimensional scaling of pollen samples based on relative abundance pollen data from the sediment cores and monoliths collected across Scilly in this study. The colors correspond with community clusters determined using a Ward's hierarchical agglomerative method (see Materials & Methods in the main text for details), which are presented in detail in Tables S3–S6. The boxplots show the distribution of axis 1 values within individual cluster communities and correspond with the same shown in Fig. 3 of the main text. The circled clusters represent pollen samples that have been influenced by marine processes and that were subsequently screened prior to developing the landcover change index.

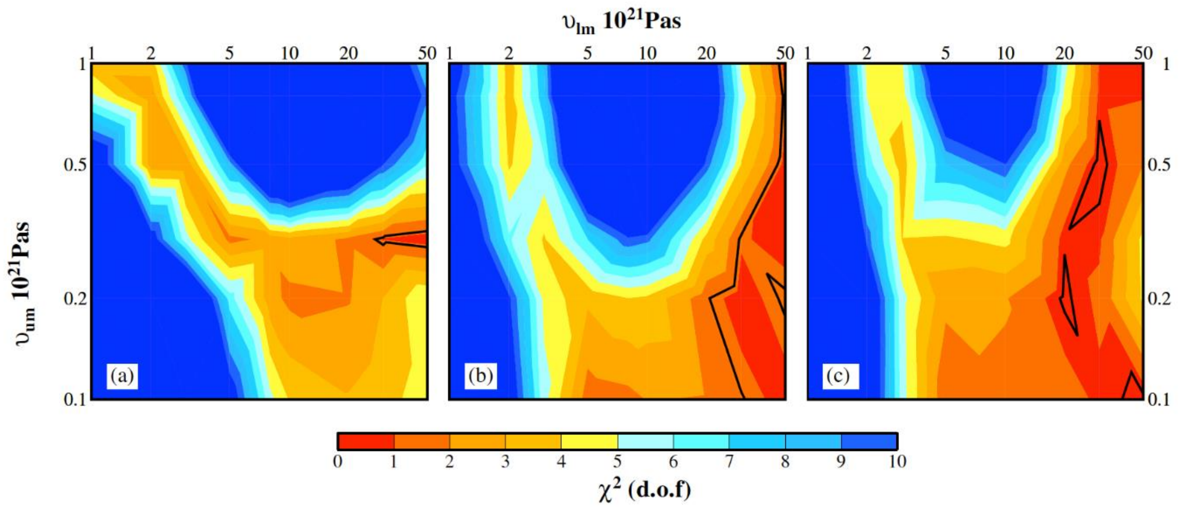

Fig. S2. Glacial isostatic adjustment modelling misfit plots. Contour plots of the normalized χ^2misfits between model predicted and observed sea-level as functions of upper (υ_{um}) and lower (υ_{lm}) mantle viscosities. Misfits for the (a) ICE5G and (b) Bradley2017 ice-sheet history models with 71 km lithosphere thicknesses and (c) misfits for the Bradley2017 ice-sheet history model with 96 km lithosphere thickness. The solid black line marks the 95% confidence limits for each model, equating to χ^2= 1.15 (a), 1.27 (b), and 0.59 (c), respectively.

(below) **Fig. S3. Archeological monument data for Scilly.** (a) Probabilistic archaeological index of population variability on Scilly (see Materials & Methods in the main text for details), constructed from the monument database (b) for Scilly from the Cornwall and Scilly Historic Environment Record. (c) Locations of Bronze Age archaeological monuments from the database overlaid on the 4 ka BP paleogeography configuration of the islands from this study.

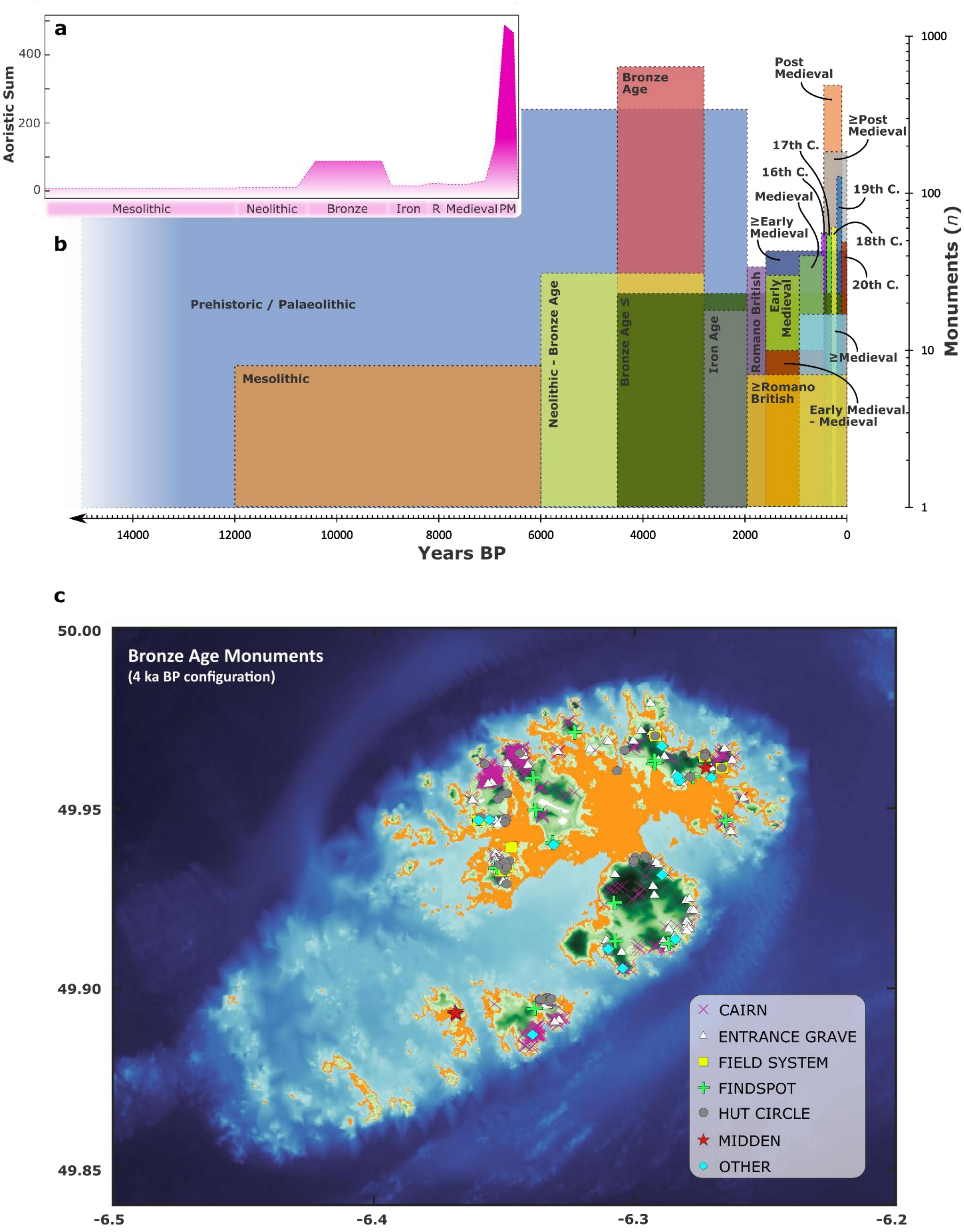

a
Aoristic Sum
400
200
0
Mesolithic
Neolithic
Bronze
Iron
R
Medieval
PM
b
Prehistoric / Palaeolithic
Mesolithic
Neolithic - Bronze Age
Bronze Age
Bronze Age ≤
Iron Age
Romano British
≥Romano British
Early Medieval
≥Early Medieval
Medieval
16th C.
17th C.
Post Medieval
≥Post Medieval
19th C.
18th C.
20th C.
≥Medieval
Early Medieval - Medieval
1000
100
10
1
Monuments (n)
14000
12000
10000
8000
6000
4000
2000
0
Years BP
c
Bronze Age Monuments
(4 ka BP configuration)
50.00
49.95
49.90
49.85
-6.5
-6.4
-6.3
-6.2
CAIRN
ENTRANCE GRAVE
FIELD SYSTEM
FINDSPOT
HUT CIRCLE
MIDDEN
OTHER

Supplementary Tables

Table S1. Summary of sediment cores and hand-cut monoliths from Scilly used in this study, with location (decimal longitude/latitude), elevation above local Ordnance Datum St Mary's and analytical procedures (C – radiocarbon dated; O – optically stimulated luminescence dated; p – pollen and landcover analyses; f – contained salt-marsh foraminifera; ch – used in the Composite Charcoal Curve).

**ID** (location)	**Original Source**	**Longitude**	**Latitude**	**Elevation** (m OD)	**Analysis**
LPTR1 (Tresco)	Charman et al. (2016)	-6.32583	49.9437	-0.03	*C,O,p,f*
LPTR2 (Tresco)	Charman et al. (2016)	-6.32647	49.94382	1.3	*C,p*
LPTR3 (Tresco)	Charman et al. (2016)	-6.33075	49.94166	0.83	*C,O,p*
LPTR4 (Tresco)	Charman et al. (2016)	-6.32568	49.94408	1.84	*C,O,p,f*
LPPM1 (Porth Mellon)	Charman et al. (2016)	-6.30914	49.91806	-1.5	*C,O,p,f,ch*
LPPM2 (Porth Mellon)	Charman et al. (2016)	-6.30964	49.91821	-2.3	*C,O,p,ch*
LPPM3 (Porth Mellon)	Charman et al. (2016)	-6.30893	49.9178	-0.82	*O,p,ch*
LPPH1 (Porth Hellick)	Charman et al. (2016)	-6.28402	49.91564	-1.27	*C,O,p,ch*
LPSM1 (St Martin's)	Charman et al. (2016)	-6.27462	49.959	-1.44	*C,p,ch*
LPSM2 (St Martin's)	Charman et al. (2016)	-6.27914	49.95863	-1.39	*C,p,f,ch*
LPSA1 (Porth Coose)	Charman et al. (2016)	-6.34959	49.89617	1.26	*C,p,f,ch*
LPOT1 (Old Town Bay)	Charman et al. (2016)	-6.30082	49.91216	2.28	*C,O,p,ch*
IMAG1-3 (St Mary's Road)	Charman et al. (2016)	-6.31548	49.93633	-7.07	*C,p*
SA11-1 (St Mary's Road)	Charman et al. (2016)	-6.26494	49.95737	-8.91	*C,p,f,ch*
SA11-2 (St Mary's Road)	Charman et al. (2016)	-6.26508	49.95738	-9.21	*C,p,f*
SA11-3 (St Mary's Road)	Charman et al. (2016)	-6.26506	49.95753	-9.11	*C,p,f*
SA2-5/1 (St Mary's Road)	Charman et al. (2016)	-6.31562	49.93518	-8.41	*C*
SA2-5/2 (St Mary's Road)	Charman et al. (2016)	-6.31564	49.93517	-8.31	*C,p,f,ch*
SA2-5/3 (St Mary's Road)	Charman et al. (2016)	-6.31559	49.93526	-8.31	*C*
SA2-5/5 (St Mary's Road)	Charman et al. (2016)	-6.3165	49.93575	-8.41	*C,p,ch*
SA2-7 (St Mary's Road)	Charman et al. (2016)	-6.31659	49.93776	-6.41	*p,f,ch*
SA2-8 (St Mary's Road)	Charman et al. (2016)	-6.31659	49.93776	-6.41	*C,p,f,ch*
HM1016 (St Mary's)	Perez et al. (2015)	-6.28643	49.91752	2.74	*C,p,ch*
LM1028 (St Mary's)	Perez et al. (2015)	-6.32583	49.9437	2.15	*C,O,p,ch*
LM1019 (St Mary's)	Perez et al. (2015)	-6.30658	49.92047	3.49	*C,p,ch*
PLOO (St Mary's)	Perez et al. (2015)	-6.30848	49.92116	2.63	*C,p,ch*

Table S2. Summary of sample results from optically stimulated luminescence dating, including age estimations that are used in this study. *Sample identifiers correspond with the English Heritage Research Report Series no.2-2013 technical report (Roberts and Marshall, 2013), where full metadata for the samples are freely available.

Core ID	Sample identifier*	Depth in core (m)	Material (grain size; µm)	No. aliquots (measured/ used)	Equivalent dose D_e (Gy)	Total dose rate (Gy/ka)	OSL age (yrs before 2010 CE)
LPTR1	161/LPTR-1	0.07-0.09	Quartz (180-210)	21/24	10.86±0.26	3.25±0.17	3340±190
LPTR3	161/LPTR3-1	0.12-0.14	Quartz (180-210)	18/24	10.43±0.30	3.96±0.20	2630±150
LPPM1	161/LPPM1-1	0.15-0.17	Quartz (180-210)	21/24	14.78±3.69	3.11±0.16	4750±1210
LPPM2	184/LPPM-2	0.09-0.13	Quartz (180-210)	24/24	11.27±0.22	2.43±0.12	4630±250
LPPM3	184/LPPM-3A	0.02-0.04	Quartz (180-210)	24/24	13.81±0.44	3.35±0.17	4120±250
LPPM3	184/LPPM-3B	0.15-0.19	Quartz (180-210)	24/24	13.17±0.31	3.07±0.16	4290±250
LPOT1	184/LPOT-1A	0.14-0.16	Quartz (212-355)	24/24	4.57±0.15	3.27±0.18	1400±90
LPPH1	184/LPPH-1A	0.15-0.17	Quartz (180-210)	24/24	34.90±0.80	2.69±0.13	12970±690
LPTR4	184/LPTR-4A	0.04-0.06	Quartz (180-210)	24/24	6.61±0.19	3.49±0.18	1890±110
LPTR4	184/LPTR-4B	0.18-0.20	Quartz (180-210)	24/24	4.57±0.27	3.32±0.18	1380±110
LM1028	184/LM10-28-161	1.61-1.65	Quartz (63-90)	24/24	4.48±0.26	1.49±0.16	3000±370
LM1028	184/LM10-28-217	2.17-2.21	Quartz (63-90)	24/24	3.89±0.11	1.28±0.11	3050±280
LM1028	184/LM10-28-277	2.71-2.77	Quartz (63-90)	24/24	5.25±0.15	1.68±0.22	3130±410
LPTR1	161/LPTR1-M	modern	Quartz (180-210)	21/24	0.007±0.006	2.85±0.16	3±2
LPTR3	161/LPTR3-M	modern	Quartz (180-210)	20/24	0.009±0.008	3.58±0.19	3±2

Table S3. Phytosociological classification results for community clusters representing intertidal and coastal environments. Pollen relative abundance data have been reduced down to genera and family orders for clarity. Frequency classes (see Materials and Methods in the main text for details) are shown in Roman numerals (low frequency taxa (I) are omitted for clarity) alongside average relative abundance values. Cluster numbers correspond with the sample groups shown in Fig. S1

**Cluster 1** (*n*=22)		**Cluster 9** (*n*=6)		**Cluster 10** (*n*=10)	
Chenopodiaceae	V (44.97)	Brassicaceae	V (48.73)	Cyperaceae	V (25.64)
Spergularia	V (19.35)	Poaceae und.	V (14.54)	Chenopodiaceae	V (17.97)
Poaceae und.	V (12.98)	Chenopodiaceae	V (12.62)	Poaceae und.	V (17.84)
Pteridium	V (10.3)	Lactuceae	V (10.52)	Plantago lanceolata	V (4.17)
Plantago lanceolata	V (4.13)	Spergularia	V (5.54)	Calluna	V (3.78)
Plantago coronopus	V (2.82)	Pteridium	V (3.21)	Plantago m/m	V (3.77)
Brassicaceae	V (1.99)	Pinus	V (1.61)	Lactuceae	V (2.49)
Quercus	V (1.93)	Plantago und.	V (1.28)	Pteridium	V (1.33)
Corylus	V (1.24)	Cyperaceae	V (0.78)	Apiaceae	V (1.24)
Betula	V (1)	Pteropsida	V (0.55)	Potentilla	V (0.72)
Armeria Type A	IV (2.18)	Plantago lanceolata	IV (0.61)	Plantago maritima	IV (5.71)
Armeria Type B	IV (1.36)	Quercus	IV (0.55)	Ranunculaceae und.	IV (2.8)
Alnus	IV (0.64)	Plantago coronopus	III (0.83)	Rumex acetosa	IV (1.27)
Plantago m/m	III (0.76)	Caryophyllaceae	III (0.22)	Prunus spp	IV (1.25)
Polypodium	III (0.71)	Calluna	III (0.22)	Rhinanthus type	IV (1.24)
Cyperaceae	III (0.59)	Alnus	II (0.22)	Cirsium	IV (0.9)
Lactuceae	III (0.38)	Corylus	II (0.22)	Anthemis	IV (0.81)
Caryophyllaceae	III (0.29)	Polypodium	II (0.22)	Corylus	IV (0.77)
Pinus	III (0.29)	Armeria Type A	II (0.22)	Pinus	IV (0.74)
Pteropsida	III (0.27)	Sedum type	II (0.22)	Myriophyllum und.	IV (0.34)
Triglochin	II (0.91)	Osmunda	II (0.17)	Galium type	IV (0.3)
Apiaceae	II (0.26)	Rosaceae	II (0.17)	Pteropsida	IV (0.03)
Filipendula	II (0.23)	Lamiaceae	II (0.11)	Poaceae >37 um	III (0.6)
Poaceae >37 um	II (0.22)			Alnus	III (0.44)
Asteroideae	II (0.21)			Rubus type	III (0.36)
Umbilicus rupestris	II (0.18)			Sphagnum	III (0.34)
Rumex und.	II (0.18)			Ericales	III (0.33)
Ranunculaceae und.	II (0.1)			Caryophyllaceae	III (0.31)
Ericales	II (0.08)			Rosaceae	III (0.3)
Plantago maritima	II (0.06)			Filipendula	III (0.3)
				Primula T.	III (0.26)
				Polypodium	III (0.24)
				Quercus	III (0.22)
				Sorbus type	III (0.19)
				Valeriana type	II (0.13)
				Brassicaceae	II (0.13)
				Asteroideae	II (0.11)
				Thalictrum	II (0.08)
				Salix	II (0.07)
				Betula	II (0.06)

Table S4. Phytosociological classification results for community clusters representing woodland environments. Pollen relative abundance data have been reduced down to genera and family orders for clarity. Frequency classes (see Materials and Methods in the main text for details) are shown in Roman numerals (low frequency taxa (I) are omitted for clarity) alongside average relative abundance values. Cluster numbers correspond with the sample groups shown in Fig. S1.

**Cluster 2** (*n*=25)		**Cluster 5** (*n*=31)		**Cluster 6** (*n*=4)		**Cluster 7** (*n*=21)	
Corylus	V (50.52)	Quercus	V (31.58)	Quercus	V (88.86)	Betula	V (73.99)
Quercus	V (19.81)	Pteropsida	V (29.02)	Pteropsida	V (4.95)	Poaceae und.	V (9.42)
Hedera	V (5.99)	Cyperaceae	V (18.56)	Betula	V (4.13)	Pteropsida	V (8.21)
Poaceae und.	V (5.36)	Betula	V (15.71)	Corylus	V (2.61)	Quercus	V (4.24)
Betula	V (4.6)	Corylus	V (12.42)	Cyperaceae	V (2.03)	Corylus	V (4.2)
Polypodium	V (2.36)	Poaceae und.	V (7.69)	Salix	V (1.39)	Salix	V (1.22)
Pteropsida	V (1.2)	Salix	V (6.24)	Polypodium	IV (0.31)	Alnus	V (0.72)
Pteridium	IV (2.87)	Polypodium	V (2.09)	Hedera	III (0.22)	Sphagnum	IV (8.61)
Chenopodiaceae	IV (2.7)	Pinus	V (0.67)	Pinus	II (0.21)	Calluna	IV (1.93)
L. periclymenum	IV (0.85)	Asteroideae	III (1.13)	Pteridium	II (0.16)	Cyperaceae	IV (1.09)
Pinus	IV (0.83)	Brassicaceae	III (0.58)	Caryophyllaceae	II (0.08)	Pteridium	IV (0.75)
Alnus	IV (0.7)	Pteridium	III (0.54)	Ericales	II (0.08)	Pinus	III (0.34)
Calluna	IV (0.53)	Hedera	III (0.47)	Calluna	II (0.08)	Chenopodiaceae	II (0.54)
Cyperaceae	III (2.35)	Plantago lanceolata	III (0.43)	Plantago coronopus	II (0.08)	Plantago coronopus	II (0.27)
Plantago und.	III (0.4)	Sparganium/Typha	III (0.35)	Poaceae und.	II (0.08)	Polypodium	II (0.25)
Plantago lanceolata	II (1.26)	Ranunculaceae und.	II (0.61)	Ulmus	II (0.08)	Ericales	II (0.16)
Salix	II (0.47)	Lactuceae	II (0.51)	L. periclymenum	II (0.07)	Ulmus	II (0.14)
Plantago coronopus	II (0.32)	Rumex und.	II (0.32)			Hedera	II (0.11)
Rosaceae	II (0.25)	Plantago und.	II (0.31)			Brassicaceae	II (0.08)
Lactuceae	II (0.25)	Caryophyllaceae	II (0.25)				
Potentilla	II (0.25)	Ulmus	II (0.22)				
Ulmus	II (0.17)	Lamiaceae	II (0.21)				
Carpinus	II (0.14)	Alnus	II (0.16)				
Tilia	II (0.12)	L. periclymenum	II (0.15)				
Apiaceae	II (0.08)	Succisa	II (0.14)				
		Filipendula	II (0.08)				

Table S5. Phytosociological classification results for community clusters representing open-ground environments. Pollen relative abundance data have been reduced down to genera and family orders for clarity. Frequency classes (see Materials and Methods in the main text for details) are shown in Roman numerals (low frequency taxa (I) are omitted for clarity) alongside average relative abundance values. Cluster numbers correspond with the sample groups shown in Fig. S1.

**Cluster 3** (*n*=324)		**Cluster 4** (*n*=14)		**Cluster 8** (*n*=87)	
Poaceae und.	V (42.85)	Cyperaceae	V (61.64)	Poaceae und.	V (23.14)
Cyperaceae	V (10.97)	Betula	V (9.28)	Betula	V (14.88)
Plantago lanceolata	V (5.7)	Poaceae und.	V (7.34)	Plantago lanceolata	V (12.76)
Corylus	V (2.8)	Quercus	V (5.38)	Corylus	V (12.36)
Chenopodiaceae	V (2.2)	Corylus	V (4.2)	Quercus	V (6.36)
Quercus	V (2.07)	Pteropsida	V (4.11)	Cyperaceae	V (3.21)
Lactuceae	V (1.99)	Salix	V (3.13)	Calluna	V (2.21)
Myriophyllum undiff	IV (4.57)	Pteridium	V (2.13)	Chenopodiaceae	V (1.6)
Calluna	IV (3.14)	Chenopodiaceae	IV (0.94)	Pteropsida	V (1.45)
Ranunculaceae und.	IV (2.35)	Pinus	IV (0.86)	Asteroideae	V (1.18)
Pteropsida	IV (2.19)	Alnus	IV (0.53)	Alnus	V (1.08)
Rumex acetosa	IV (1.75)	Ulmus	IV (0.27)	Plantago coronopus	IV (7.05)
Betula	IV (1.53)	Plantago lanceolata	III (0.89)	Ranunculaceae und.	IV (1.34)
Asteroideae	IV (1.44)	Plantago m/m	III (0.6)	Polypodium	IV (1.28)
Potentilla	IV (1.44)	Calluna	III (0.32)	Caryophyllaceae	IV (0.74)
Apiaceae	IV (0.87)	Polypodium	III (0.24)	Potentilla	IV (0.68)
Caryophyllaceae	IV (0.74)	Sphagnum	II (0.28)	Brassicaceae	IV (0.58)
Brassicaceae	IV (0.7)	Rumex acetosa	II (0.27)	Rumex acetosa	IV (0.57)
Alnus	IV (0.65)	Ranunculaceae und.	II (0.27)	Pteridium	III (1.43)
Plantago m/m	III (2.63)	Sparganium/Typha	II (0.19)	Rumex und.	III (0.65)
Plantago maritima	III (1.54)	Lythrum salicaria	II (0.19)	Lactuceae	III (0.63)
Sphagnum	III (0.8)	Lonicera periclymenum	II (0.18)	Rosaceae	III (0.45)
Galium type	III (0.7)	Plantago und.	II (0.17)	Ericales	III (0.4)
Pteridium	III (0.68)	Filipendula	II (0.16)	Hedera	III (0.38)
Rumex und.	III (0.65)	Apiaceae	II (0.16)	Apiaceae	III (0.35)
Ericales	III (0.57)	Hedera	II (0.12)	Gentianella campestris	III (0.33)
Filipendula	III (0.57)	Tilia	II (0.12)	Hydrocotyl vulgaris	III (0.32)
Cirsium	III (0.57)	Fraxinus	II (0.09)	Sphagnum	III (0.32)
Poaceae >37 um	III (0.51)	Sorbus type	II (0.07)	Pinus	III (0.21)
Pinus	III (0.5)	Asteroideae	II (0.07)	Plantago m/m	II (1.33)
Rosaceae	III (0.42)	Caryophyllaceae	II (0.05)	Cirsium	II (0.25)
Polypodium	III (0.4)			Scrophulariaceae und.	II (0.24)
Plantago coronopus	II (0.94)			Filipendula	II (0.23)
Sanguisorba T.	II (0.55)			Potamogeton	II (0.22)
Sagina type	II (0.49)			Sorbus type	II (0.22)
Prunus spp	II (0.47)			Rumex acetosella	II (0.21)
Typha	II (0.42)			Salix	II (0.2)
Salix	II (0.42)			Umbilicus rupestris	II (0.19)
Valeriana type	II (0.25)			Fraxinus	II (0.15)
Lathyrus T	II (0.17)			Ulmus	II (0.14)
Anthemis	II (0.16)			Solanum dulcamara	II (0.13)
Hedera	II (0.16)			Lamiaceae	II (0.12)
Fraxinus	II (0.16)			Cardueae	II (0.1)
Rhinanthus type	II (0.12)			Lotus type	II (0.08)
Thalictrum	II (0.11)				
Sorbus type	II (0.11)				

Table S6. Phytosociological sub-cluster classifications for community cluster 3 (Table S5).

**Cluster 3.1** (*n*=32)		**Cluster 3.2** (*n*=110)		**Cluster 3.3** (*n*=49)		**Cluster 3.4** (*n*=131)	
Poaceae und.	V (42.9)	Poaceae und.	V (37.21)	Poaceae und.	V (63.57)	Poaceae und.	V (39.78)
Cyperaceae	V (18.36)	Cyperaceae	V (19.98)	Plantago lanceolata	V (5.25)	Myriophyllum	V (6)
Pteropsida	V (12.8)	Plantago lanceolata	V (8.01)	Calluna	V (2.97)	Calluna	V (5.69)
Chenopodiaceae	V (9.42)	Myriophyllum	V (6.15)	Corylus	V (2.46)	Plantago lanceolata	V (5.34)
Quercus	V (8.63)	Ranunculaceae und.	V (5.39)	Chenopodiaceae	V (0.82)	Cyperaceae	V (5.2)
Betula	V (7.5)	Asteroideae	V (3.08)	Alnus	V (0.75)	Plantago m/m	V (4.59)
Corylus	V (5.83)	Lactuceae	V (2.32)	Potentilla	IV (2.74)	Rumex acetosa	V (3.23)
Pteridium	V (3.2)	Corylus	V (1.76)	Quercus	IV (1.72)	Corylus	V (3.07)
Pinus	V (1.32)	Quercus	V (1.45)	Cyperaceae	IV (1.71)	Lactuceae	V (2.54)
Salix	IV (1.18)	Caryophyllaceae	V (1.2)	Lactuceae	IV (1.08)	Potentilla	V (2.29)
Alnus	IV (0.76)	Betula	V (1.03)	Sphagnum	III (2.96)	Chenopodiaceae	V (2)
Ulmus	IV (0.52)	Brassicaceae	V (0.84)	Plantago m/m	III (1.77)	Apiaceae	V (1.48)
Sphagnum	III (0.71)	Plantago coronopus	IV (2.05)	Betula	III (1.15)	Pteropsida	V (1.45)
Calluna	III (0.57)	Sagina type	IV (1.21)	Rumex acetosa	III (0.83)	Quercus	V (1.15)
Hedera	III (0.32)	Rumex und.	IV (1.08)	Apiaceae	III (0.45)	Galium type	V (1.14)
Fraxinus	III (0.31)	Rumex acetosa	IV (0.93)	Ericales	III (0.35)	Cirsium	V (1.1)
Polypodium	III (0.25)	Pteropsida	IV (0.92)	Cirsium	III (0.35)	Filipendula	V (1.09)
Tilia	III (0.14)	Chenopodiaceae	III (0.99)	Pteropsida	III (0.19)	Alnus	V (0.86)
Plantago lanceolata	II (0.21)	Calluna	III (0.98)	Plantago maritima	II (1.98)	Caryophyllaceae	V (0.74)
Plantago und.	II (0.19)	Apiaceae	III (0.53)	Plantago coronopus	II (1.53)	Plantago maritima	IV (2.91)
Caryophyllaceae	II (0.16)	Lamiaceae	III (0.52)	Galium type	II (0.99)	Ranunculaceae und.	IV (1.21)
Rumex und.	II (0.13)	Pinus	III (0.4)	Prunus spp	II (0.68)	Ericales	IV (1)
		Poaceae >37 um	III (0.4)	Poaceae >37 um	II (0.45)	Brassicaceae	IV (0.93)
		Umbilicus rupestris	III (0.37)	Pinus	II (0.43)	Poaceae >37 um	IV (0.75)
		Scrophulariaceae und.	III (0.34)	Pteridium	II (0.39)	Polypodium	IV (0.69)
		Rosaceae	III (0.34)	Asteroideae	II (0.37)	Sphagnum	IV (0.68)
		Alnus	III (0.31)	Myriophyllum	II (0.32)	Betula	IV (0.65)
		Ericales	III (0.31)	Rosaceae	II (0.29)	Rosaceae	IV (0.63)
		Filipendula	III (0.28)	Valeriana type	II (0.26)	Valeriana type	IV (0.48)
		Plantgo m/m	II (1.45)	Filipendula	II (0.23)	Asteroideae	III (0.84)
		Sparganium/Typha	II (0.82)	Brassicaceae	II (0.2)	Typha	III (0.8)
		Salix	II (0.52)	Ranunculaceae und.	II (0.2)	Sanguisorba T.	III (0.78)
		Pteridium	II (0.5)	Fraxinus	II (0.19)	Rumex und.	III (0.64)
		Plantago und.	II (0.45)	Hedera	II (0.17)	Pinus	III (0.41)
		Rumex acetosella	II (0.39)	Polypodium	II (0.16)	Lathyrus T	III (0.37)
		Armeria Type B	II (0.34)	Caryophyllaceae	II (0.14)	Pteridium	III (0.33)
		Hydrocotyl vulgaris	II (0.31)	Anthemis	II (0.14)	Anthemis	III (0.25)
		Potentilla	II (0.26)	Lathyrus T	II (0.13)	Salix	III (0.23)
		Gentianella camp.	II (0.25)			Sorbus type	III (0.21)
		Succisa	II (0.23)			Hedera	III (0.21)
		Polypodium	II (0.21)			Prunus spp	II (0.89)
		Cirsium	II (0.2)			Litorella uniflora	II (0.57)
		Potamogeton	II (0.18)			Empetrum	II (0.38)
		Thalictrum	II (0.16)			Urtica type	II (0.3)
		Rhinanthus type	II (0.15)			Fraxinus	II (0.21)
		Armeria Type A	II (0.15)			Viburnum	II (0.21)
		Anthemis	II (0.12)			Rubus type	II (0.19)
		Polygonum aviculare	II (0.1)			Sagina type	II (0.18)
		Galium type	II (0.09)			Primula T.	II (0.16)
						Thalictrum	II (0.15)
						Rhinanthus type	II (0.14)
						Hypericum perf.	II (0.13)
						L. periclymenum	II (0.12)
						Fagus	II (0.1)
						Artemisia	II (0.07)

Supplementary Datasets

Dataset S1 (separate file). Relative sea-level database for Scilly comprising directly dated radiocarbon and optically stimulated luminescence samples with corresponding metainformation (lithostratigraphy, elevation, depositional environment and indicative meaning interpretations, paleotidal range change and sea-level calculations) following the 'HOLSEA' ('Geographic Variability of Holocene Relative Sea Level') protocol (Khan et al., 2019*).

Dataset S2 (separate file). Table containing pollen results as relative abundance (genus level), modelled ages and age uncertainty for pollen samples, landcover index results (community cluster numbers and nMDS ordination axes 1 and 2), foraminifera results as species counts and transfer function results as paleomarsh elevations with uncertainty (1σ). Foraminifera samples with low test concentrations have indicative ranges (from mean high water neap tides to highest astronomical tides) in place of paleomarsh elevation estimations. Foraminifera abbreviations: H.wil – *Haplophragmoides wilbertii* ; J.mac – *Jadammina macrescens* ; M.fus – *Miliammina fusca* ; P.ipo – *Polysaccammina ipohalina* ; T.inf – *Trochammina infalta* ; T.och – *Trochammina ochracea* ; A.bat - *Ammonia batavus* ; A.mam – *Asterigerinata mamilla* ; B.var – *Bolivina variablis*; E.cri – *Elphidium crispum* ; E.wil – *Elphidium Williamsoni* ; F.spp. – *Fissurina* spp. ; *Elphidium* spp. ; H.ger – *Haynesina germanica* ; L.lob – *Lobatula lobatula* ; O.spp. – *Oolha* spp. ; Q.sem – *Quinqueloculina seminula*; R.spp. – *Rosalina* spp..

Dataset S3 (separate file). Database containing three worksheets for developing archaeological indices for Scilly. 'SWBritain' – Radiocarbon dates from Devon and Cornwall used to develop a summed probability distribution curve as an estimate of population demographic variation in Southwest Britain. 'NWFrance' - Radiocarbon dates from Brittany and Normandy used to develop a summed probability distribution curve as an estimate of population demography in Northwest France. 'Scilly' – Archaeological monuments from Scilly used to develop a probabilistic index of population variability.

**Khan, N.C., Horton, B.P., Engelhart, S., Rovere, A., Vacchi, M., Ashe, E.L., Tornqvist, T.E., Dutton, A., Hijma, M.P., Shennan, I. 2019. Inception of a global atlas of sea levels since the Last Glacial Maximum. Quaternary Science Reviews, 220, 359-371.*

List of Radiocarbon Resources

The authors acknowledge the data collection and curation of radiocarbon dates into the published and online data repositories that were used in this study. Thanks to:

- Banque Nationale de Données Radiocarbone; Centere de datation par le radiocarbon (UMR 5138 CNRS); Université Claude Bernard, Lyon
  [www.arar.mom.fr/banadora/]
- Bayliss, A., Bronk, C., McCormac, G., van der Plicht, J., Cook, G. (2008) Radiocarbon dates from samples funded by English Heritage under the Aggregates Levy Sustainability Fund 2004-7. English Heritage
  [available via https://archaeologydataservice.ac.uk/archives/view/c14_cba/]
- Bayliss, A., Hedges, R., Otlet, R., Switsur, R., Walker, J. (2012) Radiocarbon dates from samples funded by English Heritage between 1981 and 1988. English Heritage
  [available via https://archaeologydataservice.ac.uk/archives/view/c14_cba/]
- Bayliss, A., Ramsey, C.B., Cook, G., McCormac, G. (2013) Radiocarbon dates from samples funded by English Heritage between 1988 and 1993. English Heritage
  [available via https://archaeologydataservice.ac.uk/archives/view/c14_cba/]
- Bayliss, A., Ramsey, C.B., Cook, G., McCormac, G., Marshall, P. (2015) Radiocarbon dates from samples funded by English Heritage between 1993 and 1998. English Heritage
  [available via https://archaeologydataservice.ac.uk/archives/view/c14_cba/]
- Bayliss, A., Ramsey, C.B., Cook, G., Marshall, P., McCormac, G., van der Plicht, J. (2017) Radiocarbon dates from samples funded by English Heritage between 1998 and 2003. English Heritage
  [available via https://archaeologydataservice.ac.uk/archives/view/c14_cba/]
- CalPal radiocarbon database; Bernhard Weninger, Cologne Radiocarbon Calibration & Paleoclimate Research Package
  [http://www.calpal-online.de/]
- Cornwall Archaeological Unit Radiocarbon Database; Cornwall Archaeological Unit, Cornwall Council, Truro
  [https://www.cornwall.gov.uk/environment-and-planning/cornwall-archaeological-unit/]
- Cornwall & Scilly Historic Environment Record; Strategic Historic Environment Service; Cornwall Council, Redruth
  [https://www.heritagegateway.org.uk/gateway/]
- European Pollen Database; Aix Marseille Université
  [www.europeanpollendatabase.net]
- Jordan, D., Haddon-Reece, D., Bayliss, A. (1994) Radiocarbon dates from samples funded by English Heritage and dated before 1981. English Heritage
  [available via https://archaeologydataservice.ac.uk/archives/view/c14_cba/]
- Manning, K., Colledge, S., Crema, E., Shennan, S., Timpson, A. (2016) The cultural evolution of Neolithic Europe. EUROEVOL Dataset 1: Sites, phases and radiocarbon data. Journal of Open Archaeology Data, 5: e2
  [dx.doi.org/10.5334/joad.100]
- Oxford Radiocarbon Accelerator Unit Radiocarbon Database
  [https://c14.arch.ox.ac.uk/database/]
- RADON radiocarbon database; Martin Hinz, Central European and Scandinavian database of 14C dates for the Neolithic and Early Bronze Age
  [https://radon.ufg.uni-kiel.de/]
